# Enhancing RAPNO: the need for standardized imaging heuristics and volumetric assessment

**DOI:** 10.1007/s00234-025-03641-x

**Published:** 2025-05-16

**Authors:** Ali Nabavizadeh, Ariana M. Familiar

**Affiliations:** 1 University of Pennsylvania, Philadelphia, USA; 2 Children’s Hospital of Philadelphia, Philadelphia, USA; 3 Children’s Hospital of Philadelphia, Philadelphia, USA

We read with interest the recent review by Geraldo et al. on the RAPNO criteria and their practical application for neuroradiologists [1]. While the authors thoughtfully outline the current limitations of RAPNO, including challenges in tumor delineation, interreader variability, and the limited role of volumetrics, we aim to build on this foundation by sharing recent efforts that offer practical strategies to address these issues and support future developments.

In a recent review [2], we proposed imaging-based heuristics for consistent identification of enhancing, non-enhancing, cystic, and edematous tumor components, offering a practical framework to guide both manual delineation and the development of automated tools. The review also emphasized the need for prospective validation of volumetric response thresholds tailored to specific tumor types and suggested subregion-specific definitions to help reduce inter-reader variability.

In our experience with multi-institutional pediatric trials, standardizing even the basic subregion definitions has proven difficult without agreed-upon imaging heuristics. This is particularly true for non-enhancing tumors and surrounding edema, which often appear indistinct on conventional sequences but require clear delineation for reproducible assessments. Response assessment can be strengthened by standardized multiparametric imaging protocols and structured annotations that support longitudinal volumetric tracking and subregion evolution [3].

Finally, while volumetric analysis remains optional in RAPNO, there is a growing consensus on its added value for irregular, infiltrative, or multifocal tumors. For such tumors, tools that offer reliable subregion tracking, change detection, and spatial mapping of new lesions over time are essential for consistent reporting and trial readiness, [4]. A natural progression from descriptive frameworks to quantitative, validated tools that are widely accessible grounded in robust imaging standards [5], is urgently needed.

We commend the authors for providing a valuable resource and believe that the next phase of RAPNO development should be driven by practical, implementation-ready strategies. These approaches can enhance consistency across centers, improve reproducibility of imaging assessments, and ultimately support patient-level decision making in both clinical trials and routine care.

## Data Availability

No datasets were generated or analysed during the current study.
